# Reminiscences of the Insane

**Published:** 1887-02-26

**Authors:** 


### Reminiscences of the Insane.

By a Mental Physician.

(*Continued from page* 337.)

Before passing on to another section of these Reminiscences, we desire to supplement the remarks made in a previous paper (see The Hospital, p. 254) in reference to the Physiognomy of the Insane.

Mental Stupor.

There is a class of cases which have frequently attracted the attention of novelists and artists. A person passes from his ordinary state of health into one of complete mental abstraction. In popular language, the mind has gone out, and yet even the non-medical observer feels that the person is not in a state of hopeless imbecility, but is wholly absorbed in some intense and overwhelming thought, which renders the outer world invisible. It may be a state of ecstasy, an absorption of the whole soul in one idea, introducing the happy and passive subject of it to a celestial vision, a subjective world, peopled with saints or angels, and accompanied by strains of heavenly music. It is a continuous condition of self-hypnotism, and resembles those states which can be temporarily induced by the hypnotist. Much more frequently, however, this intense abstraction and absorption of mind is associated with some terrible delusion, which constitutes a veritable day-mare. The patient may be impressed with the belief that to take one step forward will plunge him into the lake of brimstone, and in some instances he believes that he is either going to be delivered into the hands of tormentors, or cast into everlasing fire. In either case—the happy ecstatic or the melancholy patient—he or she stands like a statue, and the limbs may frequently be placed and retained in any position in which it is desired to fix them.

Catalepsy.

One remarkable feature of such cases is the possibly sudden return to consciousness and health, the patient relapsing frequently into the former statuesque attitude. There are other cases in which there are two distinct personalities, and two distinct lives seem to be led. The very popular fiction, entitled *Called Back*, was based upon a knowledge of some curious instances of this kind, although it cannot be said that it was altogether true to nature, or perhaps it ought rather to be said, to mental disorder. The still more extraordinary and fascinating picture which the accomplished author of *Dr. Jekyl and Mr. Hyde*, has drawn, can hardly be said to find its counterpart in asylum life, but it closely resembles the astonishing conditions of double and even treble consciousness which are sometimes induced by hypnotic means. We have seen a woman who lives three different and entirely distinct lives, in none of which does she remember what has passed in the other states of existence.

Fatuity.

In strong contrast to the physiognomical expression of patients labouring under acute excitement—active, melancholy, or delusional insanity—are the facial characters of dementia or fatuity. Here, while we have in all an absence of mental power, there are at least two forms of outward expression, characteristic of the mental loss which has overtaken the patient.

The Blank Face.

There is the absolute *blank*, the collapse of the muscles of expression, the lack-lustre eye, the inane mouth, as well as the general purposeless bearing and gait of the fatuous. Such a combination, altogether apart from any incoherence, silence, or monotonous muttering of the patient, stamps the case with the seal of dementia. The patient is reduced to an inert mass of humanity, or mindless pulp.

The Weak Face.

The other variety is not so generally associated in the mind of the ordinary observer with hopeless fatuity. Here the patient may be busily engaged throughout the day in some simple occupation of a more or less mechanical nature. He is the hewer of wood and drawer of water in our public asylums. His expression is not a blank, but represents general contentment, or even fussy activity. That his face shews lines of feebleness when compared with that of his prime, is very true, but this variety of mental decay might not reveal the true condition of the patient to a stranger, without conversation with the patient. An experienced observer would no doubt be more likely to detect the real mental condition, even in the mask which, by retaining certain indelible features of a former self, conceals the vacuity beneath; but even he, be his eye ever so practised, might greatly err in his physiognomical readings, in the absence of conversation, observation of the patient's acts, and a knowledge of his previous history.

Excitement with Fatuity.

There is, likewise, the expression of chronic mental weakness which is associated with attacks of excitement, and here we have to do with a numerous class of cases which insensibly glide into those of chronic mania; in fact, they may be indistinguishable, inasmuch as the patient who suffers from prolonged excitement or mania is very likely to pass into incurable weakness of mind; while, on the other hand, those patients who are properly called "dements," are subject to violent impulses, just as the calm surface of the lake becomes agitated by a sudden squall.

Primary Dementia.

We have spoken of Mental Stupor. It is often hard to say whether in this condition there is an all-absorbing delusion which reduces the sufferer to an apparently mindless state, or whether there is a real loss of mental capacity, including memory, and the recognition of the friends and surroundings of the patient. That there may be, from shock or exhaustion, a mental blank which for the time being constitutes dementia, cannot be questioned. The facial expression is then that which we have already described as belonging to fatuity. A blow on the head may induce this condition. There cannot in this case be any terrible delusion which prevents the patient manifesting his usual mental faculties. His brain has received an injury which absolutely prevents the exercise of its functions, and no one can say whether it will recuperate or not. Mental physicians, for the sake of convenience, call this a state of Primary Dementia, to distinguish it from the dementia which succeeds to mania, or other forms of mental disease. But it is to be remarked that this Primary Dementia may present a form so little like ordinary dementia, and so much like Mental Stupor, that then it is much more convenient to give it this name, and to reserve that of "Mental Stupor with Melancholia" for the cases of overpowering delusion and of ecstasy.

Summary.

So then, the figures and faces which arise before the memory of the mental physician when he dwells for a moment on the population of the insane world in which he has moved, are composed, broadly speaking, of those patients over whom a tornado of excitement passes; those into whose very soul the bitterest misery has entered; those who are swayed by fantastic delusions; and, lastly, those—constituting, unfortunately, the great majority in our hospitals for the insane—who have lost their intelligence beyond all hope of recovery, and, leading a merely vegetative life, pass a monotonous, mechanical routine existence; or, it may be, having not only lost their intellect, but all control over their impulses, are ever and anon impelled to acts of violence, and are drawn hither and thither by gusts of passion. "To such complexion may we come."

(*To be continued.*)

